# Angioarchitecture and hemodynamics of microvascular arterio-venous malformations

**DOI:** 10.1371/journal.pone.0203368

**Published:** 2018-09-07

**Authors:** Sabrina Frey, Tarcisi Cantieni, Nicolas Vuillemin, Axel Haine, Rafael Kammer, Hendrik von Tengg-Kobligk, Dominik Obrist, Iris Baumgartner

**Affiliations:** 1 ARTORG Center for Biomedical Engineering Research, University of Bern, Bern, Switzerland; 2 Swiss Cardiovascular Center, Division of Angiology, University of Bern, Bern University Hospital, Bern, Switzerland; 3 Department of Diagnostic, Interventional and Pediatric Radiology, University of Bern, Bern University Hospital, Bern, Switzerland; Technion Israel Institute of Technology, ISRAEL

## Abstract

**Introduction:**

Arteriovenous malformations (AVMs) are characterized by pathological high flow, low resistance connections between arteries and veins. Treatment is critically dependent on correct interpretation of angioarchitectural features. However, some microfistular AVMs do not match the characteristics described in current AVM classification systems. Therefore, we propose a new subgroup of microfistular AVMs, composed of enlarged, fistulous paths on the venous half of capillaries and/or dilated draining venules (hyperdynamic, capillary-venulous malformation [CV-AVM]). CV-AVMs still ensure arterial flow to the periphery and fistulous venous drainage is less pronounced than in classical AVMs such that these lesions are often misinterpreted as venous malformations.

**Materials and methods:**

We developed a computational model to study the effects of microvascular anomalies on local hemodynamics, as well as their impact on angiographic contrast propagation. Flow rates and pressures were computed with a lumped parameter description, while contrast propagation was determined by solving the 1D advection-diffusion equation.

**Results and conclusions:**

For the newly proposed CV-AVM angioarchitecture, the computational model predicts increased arterio-venous contrast agent transit times and highly dispersive transport characteristics, compared to microfistular, interstitial type IV AVMs and high flow type II and III AVMs. We related these findings to time-contrast intensity curves sampled from clinical angiographies and found that there is strong evidence for the existence of CV-AVM.

## Introduction

Congenital vascular malformations are vascular anomalies, which arise during embryonic vascular development and represent an inborn error of the normal vascular angioarchitecture. Depending on the affected vascular systems, lesions are grouped into arterio-venous, venous, capillary or lymphatic malformations [1].

**Fig 1:**
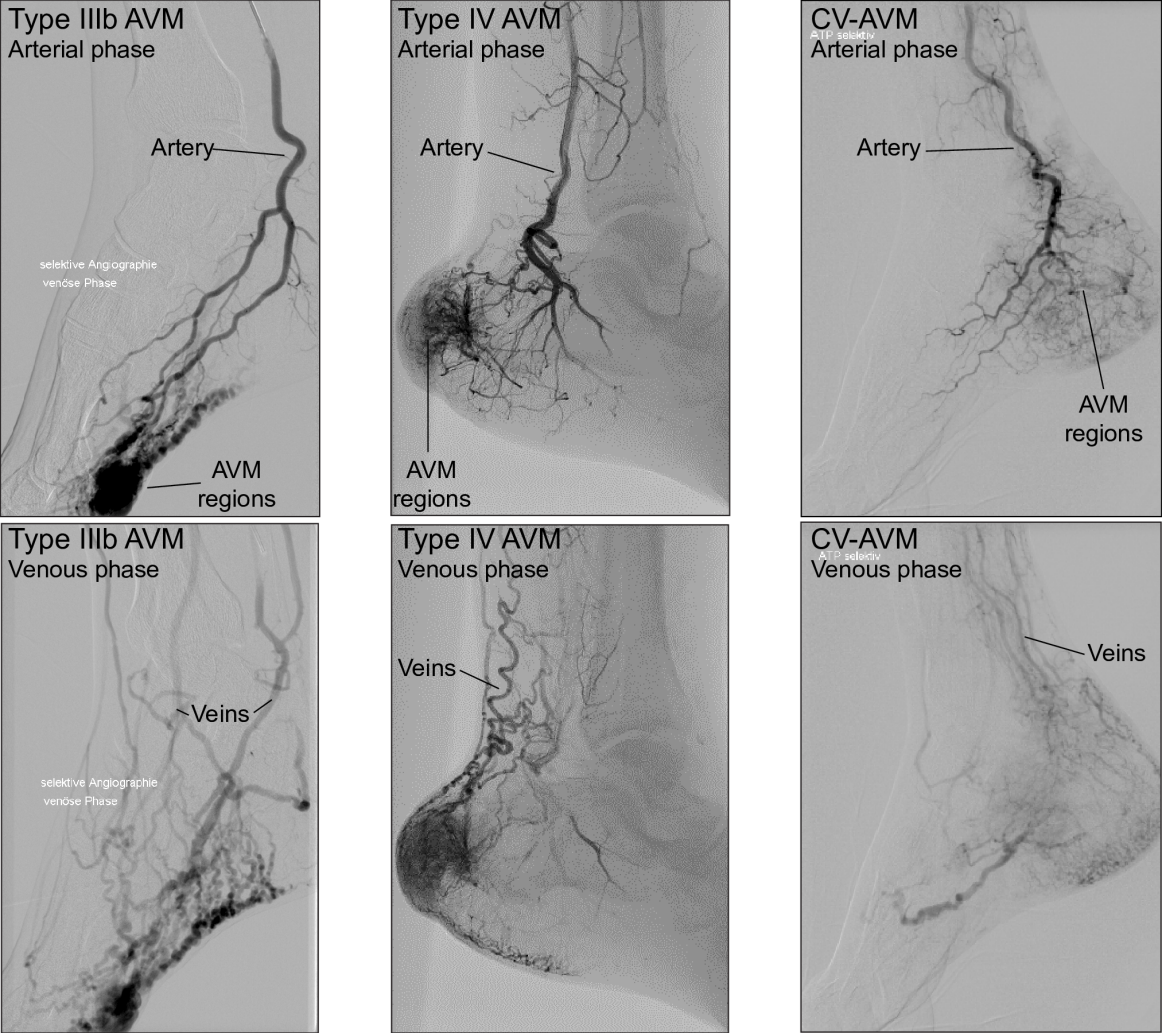
DSA recordings of AVM cases. Arterial (upper row) and venous phases (bottom row) in DSA recordings of patients diagnosed with Yakes type IIIb (left column), Yakes type IV (middle column) and hyperdynamic CV-AVMs (right column).

Arterio-venous malformations (AVMs) are anomalous networks of high flow, low resistance connections between arteries and veins, which bypass the capillary bed [2]. Beside clinical aspects, AVMs are diagnosed using duplex ultrasound to assess arterial and venous flow characteristics, non-invasive magnetic resonance (MR) imaging and catheter based intra-arterial digital subtraction angiography (DSA). For detailed AVM specification, DSA with arterial injection of contrast agent (CA) and simultaneous observation of CA spread in the malformation remains the gold standard for diagnosis (Fig 1). The presence of an AVM causes rapid spread of CA in the arterial system, as well as an early and non-dispersed venous drainage.

AVMs can be classified into different types according to their angioarchitecture. The Yakes classification [3] is a recent adjustment of the nomenclature that builds on previously reported AVM morphology descriptions [4,5]. Yakes differentiates four basic types of AVMs depending on their angioarchitecture and observed hemodynamic behavior [2]. Yakes type I AVM describes a single shunt between an artery and a vein. Type II is a complex tangle of small-sized abnormal blood vessels (nidus) with several feeding arteries and draining veins. Type IIIa/b are described as multiple feeding arteries draining via the wall of an aneurysmal vein to single/multiple outflow veins (Fig 1, left column). In type I-III AVMs, no intervening capillary bed is present. Increased venous pressures and high flow rates in the malformation often lead to a complete lack of parallel capillary perfusion (arterial steal). Yakes also reports on AVM variants with an abnormality in the microcirculation (interstitial type IV, Fig 1, middle column). Type IV AVMs are composed of anomalous connections between arterioles and venules that are interwoven with normal capillaries, which maintain the viability of surrounding tissue. Clinical presentation is similar to type I-III AVMs, although no clear angiographic nidus is visible on DSA, but rather a dense contrast blush. Moreover, hemodynamic changes are less dramatic and venous shunt flow is delayed compared to type I-III AVM [2,3].

At the other end of the spectrum are venous malformations. They are low-flow congenital malformations composed of thin-walled, dilated veins [6]. Blood flow is extremely slow and can become stagnant [7,8].

Although most malformations can be classified into one of the presented lesion types, some lesions do not present typical features and cannot be associated with a known lesion type [9]. Over the past decade, the Division of Angiology of the University Hospital Bern has treated a significant number of patients with congenital vascular malformations exhibiting characteristics of type IV AVMs together with features that are commonly associated with venous malformations. DSA recordings show diffuse, “cloudy” regions of CA accumulation similar to type IV AVMs, but venous drainage is more delayed and dispersed (Fig 1, right column). The regional accumulation of CA and partially elevated arterial flow suggests the presence of a hyperdynamic path with low vascular resistance between the arterial and venous system, although venous drainage pattern is untypical for classical high-flow AVMs.

We hypothesize that there exists a hyperdynamic, capillary-venulous (CV-AVM) subgroup of “microfistular” AVMs composed of fistulous paths on the venous half of capillaries and/or dilated draining venules (hyperdynamic, capillary-venulous malformation [CV-AVM]). The pathologic pressure gradient in CV-AVMs still ensures normal arterial flow to the periphery with fistulous venous drainage that is less pronounced compared to classical AVMs. To test the hypothesis, we developed a computational hemodynamic model that allows studying the theoretical effects of microvascular anomalies, such as CV-AVM and type IV AVMs, on local blood velocities, flow rates as well as their impact on CA propagation, with special emphasis on CA transit times and dispersion. The computational findings are compared to clinical observations to determine if the hypothesized microvascular malformation morphologies can explain the pathological CA transport patterns measured in patients.

## Materials and methods

### Microcirculation and AVM graph models

**Fig 2:**
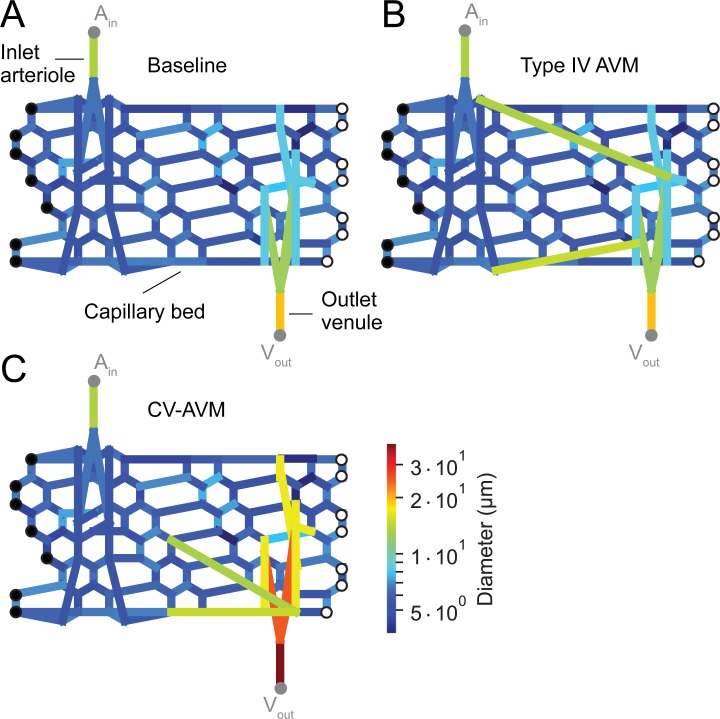
Prototype capillary bundle elements and microvascular AVMs. Schematic representation of a capillary bundle element with feeding arteriole and draining venule. (A) Baseline: physiological reference condition; (B) Type IV: example of Yakes type IV AVM; (C) CV-AVM: hypothesized morphology of a hyperdynamic CV-AVM.

A model representing the microcirculation in skeletal muscle was developed to study the effects of morphological abnormalities in the microvasculature on hemodynamic parameters and arterio-venous CA transport. Clinical observations suggest that microvascular malformations can affect large tissue regions, which cannot be represented in a computational model, due to their complexity and associated high computational costs. Therefore, we study microvascular CA transport on an isolated tissue slab, which contains a fully resolved capillary bundle element with penetrating transverse arterioles and collecting venules (Fig 2).

The arteriolar and venular trees are constructed according to average values for vessel dimensions and tree topology [10,11]. Arterial and venous distensibility values are assigned to the vessel segments according to Gamble and Bérczi [12,13]. Similarly, we build a capillary bundle element following statistical data on the connectivity of skeletal muscle microcirculation vessels introduced by Skalak et al. [11,14]. Vessel dimensions (i.e. segmental diameter and lengths) are randomly sampled from the reported distributions. Capillary distensibility is chosen according to Skalak [15], with slightly decreased/increased values for segments near the feeding arteriole and draining venule, respectively. The resulting networks contain 108 nodes and 175 segments, where 11/18 segments are part of the arteriolar/venular tree and 146 edges correspond to capillary segments.

Starting from 25 random physiological reference configurations, we introduce two types of microvascular malformation morphologies, Yakes type IV AVM (Fig 2B) and hyperdynamic CV-AVM (Fig 2C). Type IV AVMs are built by randomly sampling pairs (n ∈ {4,6,8}) of arteriolar and venular nodes from a uniform distribution. The segmental length and distensibility of these additional connections are defined as the average length and distensibility of the vessels being connected by the malformation, while their diameter corresponds to double the average diameter of the connected vessels. CV-AVMs are built by increasing the diameter of the draining venular tree (x_factor_ ∈ {2,3,4}) and introducing a set of additional connections (n ∈ {2,4,6,8}) between the arterial half of the capillary bundle and the venular nodes. These additional connections may represent the presence of abnormal connections and/or enlarged pathways in the venous capillaries. The node pairs are randomly sampled from a uniform distribution and their geometric and mechanical properties are defined following the same strategy as in type IV AVMs.

In all cases, the ranges for n and x_factor_ are chosen such that the resulting hemodynamic behavior differs substantially from the physiological reference state and such that it covers the hemodynamic parameter ranges observed in patients. The factors for venular dilation x_factor_ in CV-AVMs are additionally motivated by observations made in large scale, congenital venous anomalies [16]. Since statistical data on the morphometry of microvascular AVMs is not available, we choose equidistant samples within the limits of the ranges.

### Blood flow and substance transport modelling

Blood flow and CA transport within the microvascular networks are computed with the strategies described in our previous study on peripheral AVMs [17]. In brief, blood flow is simulated with a lumped parameter approach, where the Navier-Stokes equations [18] are averaged in space over each segment of the network (lumped element). This results in a set of ordinary differential equations in time for each segment. The segments are coupled by enforcing continuity of pressure and conservation of mass at each node of the network. We account for the non-Newtonian behavior of blood in microcirculatory networks by adjusting the apparent viscosity for vessel diameter and local hematocrit [19] using the relation by Pries [20] and empirical data by Lipowsky [21].

At the arteriolar inlet and venular outlet nodes we imposed pressure boundary conditions, which were obtained from a computational model for a physiological reference configuration [17] by probing the pressures in the pre- and post-capillary elements. The inlet pressure has a systolic/diastolic value of 60/57 mmHg, while the outlet pressure is nearly constant at approximately 13 mmHg. The left and right boundary nodes of the bundle elements (filled and empty circles in Fig 2) are connected with an additional boundary segment, which results in periodic boundary conditions and mimics an infinitely long capillary bundle with alternating penetrating arterioles and venules.

CA transport is determined by solving the 1D advection-diffusion equation on the sub-divided network, based on interpolated advection velocities and a diffusion coefficient describing binary molecular diffusion of iodinated contrast agent in blood [17]. At the CA injection site (node A_in_, Fig 2), we apply a typical temporal CA injection profile sampled from DSA recordings (cf. next section), whereas convective outflow conditions [22] are imposed at the venular outlet node (V_out_, Fig 2).

### DSA sampling

The computational results are compared to DSA recordings of patients diagnosed with a congenital microvascular AVM type IV or hyperdynamic CV-AVM. As a reference, we also include angiograms of patients diagnosed with Yakes type II and type IIIa/b AVMs. Non-pathological baseline angiograms were not included as visualizing of the venous phase after arterial CA injection in the healthy vasculature tree would require high contrast and radiation doses, because healthy tissue has many vascular pathways with slightly different path lengths, yielding very low contrast signals reaching the venous system.

All patient data were obtained from the VASCOM (VAScular COngenital Malformation) registry. All patients gave written informed consent and the study was approved by the Ethics Committee of the Canton of Bern (local ethics board number 2016–01503).

**Fig 3:**
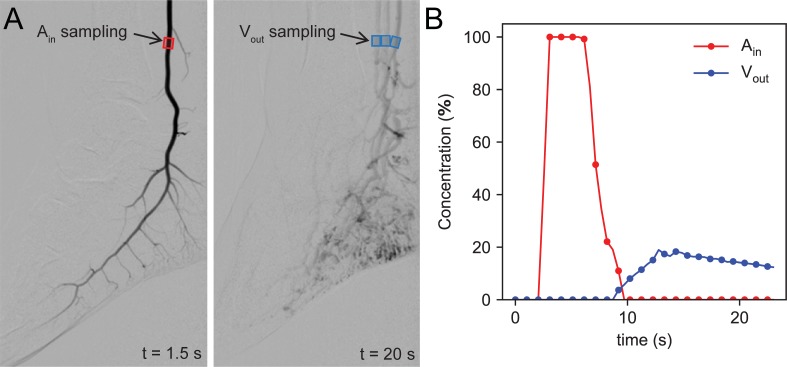
Example of concentration measurements in DSA recordings. (A) Arterial and venous sampling ROIs, (B) normalized CA intensity evolution for ROIs depicted in A.

Arterial and venous CA concentration and intensity curves are sampled at nodes A_in_ and V_out_ of the computational models and at specific locations in DSA recordings. The considered malformations are confined to the same anatomical region (foot) and all intensity curves are sampled at similar locations (0–15 cm above the lateral malleolus). For DSA sampling, we select small regions of interest (ROIs) that contain a segment of each feeding artery and draining vein (Fig 3). For each temporal frame, the intensity values contained in all arterial/venous ROIs are summed up and divided by the number of non-zero pixels. When overlapping arteries and veins are present, venous sampling is started only when the arterial signal vanishes. This way, we obtain an equivalent inlet and outlet time-concentration curve that can be used for further analysis. Note that this concentration refers to normalized pixel intensities, rather than the chemical concentration of a substance. The two quantities are proportional within the linear operating range of the image acquisition device.

### Data analysis

The comparison of computational results and *in-vivo* data is performed by means of the parameters of a transport operator (TOp) or transfer function, which translates the CA concentration curve sampled at the arterial inlet into the concentration at the venous outlet. The TOp is based on a differential operator for vascular transport phenomena introduced by King [23]. It is composed of a delay (modelling advection) and a fourth order differential operator (modelling dispersion and diffusion), which has the shape of a skewed unimodal distribution. The TOp parameters are optimized with a least-squares algorithm [24], such that the output of the TOp approaches the computed and measured venous concentration curves. The first and second central moments of the fitted TOp are used as representative parameters for the advective and dispersive behavior of the considered vasculature and will be termed arterio-venous delay t_d_ (first moment) and absolute dispersivity AD (square root of second moment) in the following.

TOps for the computational models describe transport phenomena on the microscopic scale. In contrast, TOps fitted to DSA recordings also include the dispersivity and delay introduced by the macroscopic circulation, which results in different time scales for t_d_ and AD. To compare the different TOps, we normalize t_d_ and AD with the characteristic network turnover time t_c_, defined as the ratio of the total vessel volume V_tot_ between the sampling points and the total volumetric flow rate Q_tot_ in healthy individuals. For computational models, the calculation of t_c_ is straightforward, because V_tot_ and Q_tot_ can be determined from the model description and blood flow calculations. For patient data, representative values for V_tot_ and Q_tot_ were obtained from literature [25–28].

## Results

### Hemodynamic analysis

**Fig 4:**
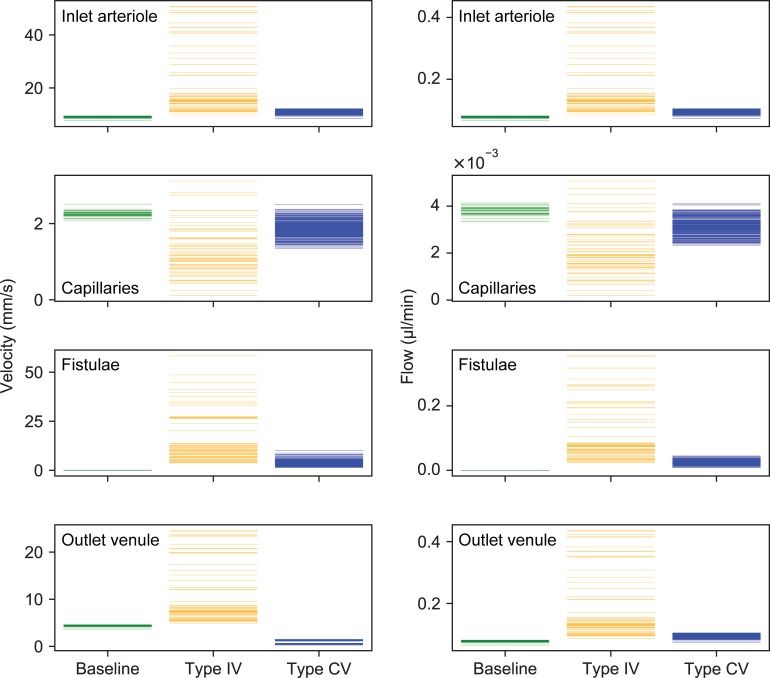
Hemodynamic parameters of computational AVM models. Temporal mean of blood flow velocities (left column) and flow rates (right column) in the inlet arteriole (first row), capillary bed (second row, mean value over all capillaries), fistulous vessels (third row, maximum value over all fistulae) and outlet venule (fourth row) for all computed prototypes. Each solid line represents one prototype baseline (green), type IV (orange) and type CV (blue) configuration.

Computed blood flow velocities and volumetric flow rates are shown in Fig 4 for all investigated physiological baseline configurations, for type IV AVMs and for hyperdynamic CV-AVMs.

For baseline networks, velocities in the inlet arteriole are in the range 7.6–9.5 mm/s and 3.6–4.6 mm/s in the outlet venule. The average velocities within the capillary bed lie in the range 2–2.5 mm/s.

A type IV AVM increases the velocity in proximal arterioles and venules, while capillary velocities and flow rates are decreased with respect to baseline conditions. The increase in arteriolar and venular flow and the reduction in capillary perfusion is stronger, the larger the number of fistulae and the more proximal the location of the shunts. Velocities and flow rates in the malformation also depend strongly on the size, location and number of shunting vessels and are 3.7–58 mm/s and 0.02–0.36 μl/min, respectively.

For hyperdynamic CV-AVMs, the inlet arteriole can exhibit increased velocity values with respect to the baseline configuration. However, outlet venule velocities are consistently smaller than in type IV and baseline networks, whereas the flow rates are mostly higher than the baseline. Capillary flow rates and velocities can be similar to the non-pathological vasculature for a small number of microfistulae, shunts feeding from distal capillary nodes and for little venular dilation. More severe malformations reduce the velocities and flow rates in the capillaries. Velocities and flow rates in the fistulous vessels are 1.4–10 mm/s and 0.008–0.04 μl/min, respectively.

### CA transport and radiographic analysis

**Fig 5:**
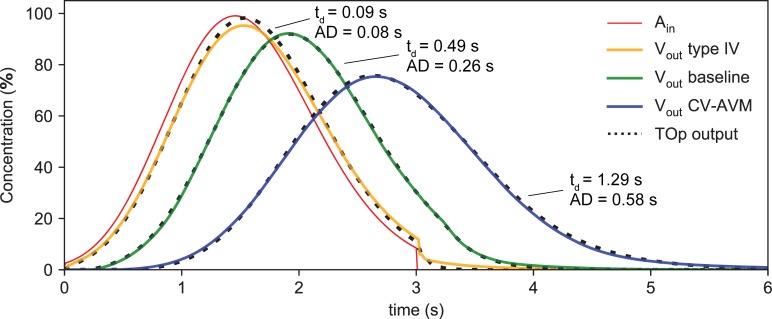
CA concentration at inlet and outlet nodes of computational AVM models. Temporal evolution of CA concentration at the injection point A_in_ (red curve) and at the venular outlet node V_out_ of computational prototypes for baseline conditions (green), type IV (orange) and CV (blue) AVMs. The dashed black lines represent the output of the fitted TOp for each configuration.

Arterio-venous transport patterns in computational and clinical results are compared to relate specific microvascular AVM morphologies to their manifestation in diagnostic DSA images. Fig 5 shows CA concentration near the arterial injection site and the resulting venous return for different types of microvascular AVMs and the baseline configuration. The curves illustrate the main effects of the malformations investigated in this study. Type IV malformations exhibit a faster and less dispersive arterio-venous CA transport than CV-AVMs, which is reflected by a smaller distance between the arterial injection profile and the venous return curve (small t_d_) and by the smaller broadening of the curve (small AD). In contrast, CV-AVMs exhibit a larger delay between arterial injection and venous return, while the venous profile-width is strongly increased (higher dispersivity).

**Fig 6:**
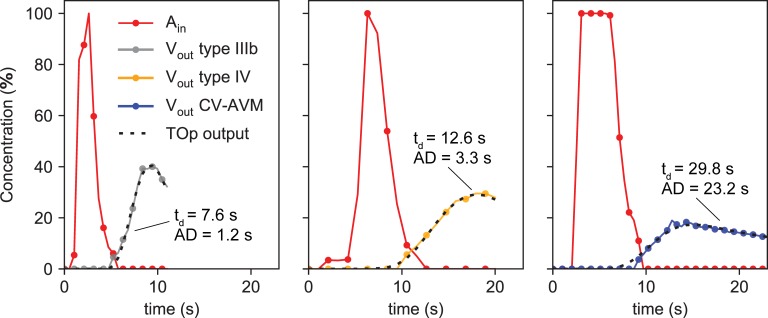
Analysis of DSA recordings of AVM patient cases. The dots on the solid curves represent sampled arterial (red) and venous (grey for type IIIb, orange for type IV and blue for CV-AVM) equivalent concentration values, while the dashed black line shows the corresponding fitted TOp output.

Fig 6 shows arterial and venous CA intensities obtained from DSA recordings of microvascular AVMs and, for comparison, of a classical type IIIb AVM. Type IIIb shows an even faster and less dispersive behavior than type IV, and the CV-AVM is again the slowest and most dispersive malformation. The DSA recordings were stopped shortly after the peaking of the venous CA return to limit radiation dosage. The classification of these cases corresponds to the existing clinical diagnosis, which is based on DSA and duplex ultrasound recordings (as described in the first section of the manuscript). Although the clinical diagnosis of microfistular AVMs with strongly delayed and dispersed venous CA appearance (CV-AVM) already exists, the morphology of these AVMs is unknown. The purpose of this study is to determine if the hypothesized angioarchitecture leads to the abnormal arterio-venous CA transport patterns observed in patients (e.g. the behavior shown in Fig 6). Note that the time scales in Figs 5 and 6 are different due to the different sampling vessels (cf. Materials and methods).

Venous return curves are highly dependent on the precise shape of the injection curves. A quantitative comparison of the venous return curves among different patients is therefore difficult. Instead, we compare the TOp parameters t_d_ and AD of different AVMs, which have been determined from the corresponding CA curves. The coefficient of determination r^2^ achieved for shown computational TOps lies above 0.98, while TOps fitted to clinical measurements yield r^2^>0.97, when applying the operator to the arterial curve and comparing the output (dashed curves in Figs 5 and 6) to the computed/measured venous curve.

**Fig 7:**
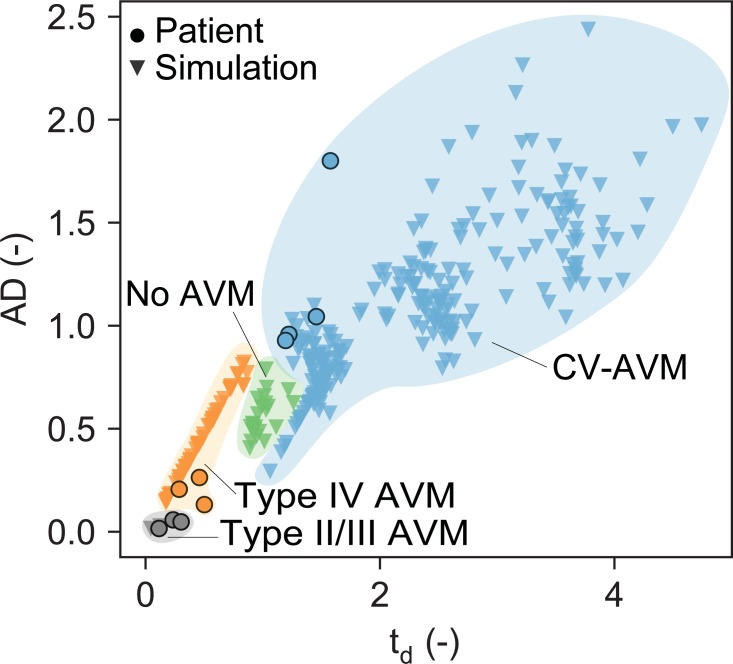
AD and t_d_ values for computational AVM models and patient cases. Scaled AD (absolute dispersion) and t_d_ (total delay) for baseline (green triangles), type IIIb (grey triangles), type IV (orange triangles), type CV (blue triangles) and type IIIb (grey triangles) computational models (n = 315) and type IV (orange circles), type CV (blue circles) and type I/III (grey circles) patient cases (n = 10).

Fig 7 shows scaled t_d_ and AD values of computational (triangles) and clinical cases (circles). Computational results (triangles in Fig 7) show that type IV AVMs exhibit AD values in the range of 0.1–0.8 and t_d_ < 1. In this case, all AD values above 0.4 (and t_d_ > 0.4) are observed in type IV models, where fistulae are either small in number or feeding from distal nodes of the arteriolar tree. CV-AVMs result in t_d_ ≥ 1 and AD ≥ 0.3. All CV-AVM cases with similar AD values as the baseline (0.3 ≤ AD ≤ 0.8) correspond to model networks with small venular dilation (i.e. x_factor_ < 3). At the other end of the parameter range, AD and t_d_ can become arbitrarily large, when increasing the diameter of the draining venules to unphysiologically large values. Baseline t_d_ values mostly lie around 1 and AD lies between 0.4–0.8. Computations by Frey et al. [17] for large scale type IIIb AVMs exhibit extremely small AD and t_d_ values around 0.02–0.03 and 0.05–0.1, respectively. *In-vivo* measurements of all malformation types (circles in Fig 7) perfectly fit into the reported computational ranges.

In summary, CV-AVMs are characterized by large t_d_ and AD values, while type IV show smaller CA dispersion and delay. Type II and III AVMs thereby show an even faster and less dispersive shunting behavior than type IV AVMs.

## Discussion

This study confirms that different types of CA transport patterns observed in AVM patients are a consequence of intrinsic differences in lesion angioarchitecture. Differences in these patterns have been quantified both with computational models and by analyzing contrast propagation in diagnostic DSA recordings. We found strong evidence for the existence of a microvascular, hyperdynamic CV-AVM, which is different from any AVM angioarchitecture described so far. Our results show that the hypothesized CV-AVM morphology of enlarged, fistulous paths on the venous half of capillaries and/or dilated draining venules accurately describes the phenomena observed in patients.

Computed blood flow velocity ranges obtained with our model of fully resolved capillary bundles with penetrating transverse arterioles and collecting venules are in good agreement with the values reported in literature. Typical velocities measured in mammal arterioles are in the range 5–10 mm/s, while venules exhibit values around 2–5 mm/s [11]. Capillary velocities attain mean values around 2–3 mm/s [29]. These ranges fully contain the computational values reported in this study. The agreement between baseline models and *in-vivo* values is crucial for the predictive capacity of the model and strengthens the validity of the results. As stated above, CA transit characteristics of the computational baseline may be suited to describe local phenomena, but cannot accurately describe the strongly dispersive macroscopic behavior of non-pathological vasculature, which consists of innumerable paths composed of similar capillary bundles with similar morphological and hemodynamic characteristics. For this reason and because of the lack of DSA recordings of healthy individuals, we restrict our analysis to the comparison of different malformation types, without comparing the results to a macroscopic physiological reference state.

The temporal evolution of CA concentration at the injection point and at the venular outlet node V_out_ of the computational prototypes shows typical type IV and CV-AVM time-concentration curves that clearly exemplify the two distinct CA transport patterns observed in both computational and *in-vivo* results. CV-AVMs show a slow and dispersive shunting behavior, while type IV AMVs are faster and remarkably less dispersive. These characteristic transport patters are a direct consequence of the strongly differing hemodynamic behavior of the two lesion types. For type IV AVMs, flow velocities in the proximal arterioles and venules are increased due to the reduced vascular resistance caused by the additional, comparatively large connections between arterioles and venules. The reduction of flow rates in the parallel capillaries is a similar effect as the arterial steal [1] observed in larger AVMs, but on a smaller scale and without compromising the viability of the surrounding tissue to the same degree, as perfusion is mostly sustained [2,3]. Even so, flow rates and velocities in the malformation can be several orders of magnitude higher than in the parallel capillary bed, which results in the observed fast and only little dispersive shunting of CA. Thus, “steal” is large enough, such that venous CA signals in type IV AVMs are mostly composed of the substance shunted through the malformation. Since the malformation is composed of a lesser number of paths than the capillary bed, dispersion is reduced. Additionally, the high velocities in the shunt result in small CA advection times.

For hyperdynamic CV-AVMs, the overall vascular resistance is decreased due to the presence of fistulae in the capillary bed and the postulated enlargement of venules, which leads to the increase in arteriolar velocities and flow rates. However, this effect is not as pronounced as in type IV AVMs. Similarly, the presence of fistulae in the venous half of the capillary bed reduces parallel capillary perfusion less strongly than in type IV lesions, which again ensures the viability of surrounding tissue, a phenomenon also observed in patients. Fistulae in the venous capillaries, though also representing a preferred, low resistance path for blood and CA, act in a similar temporal and spatial scale as the parallel capillaries, which results in small “steal” and increases dispersion since venous CA signals are composed of both CA transported through healthy and diseased vessels (additional path length differences). The strong enlargement of venules, though causing an increase in volumetric blood flow rates (decreased resistance), decreases the velocity in these vessels, amplifying dispersivity, while also increasing advection time.

These observations clearly match the clinical presentations of type IV and CV-AVMs. The former is characterized by increased arterial and venous flow rates and velocities, while the latter only shows little increase in arterial flow and a decrease in venous flow velocities due to the enlarged venous diameter. The strikingly slow and dispersed venous drainage of CA in CV-AVMs is the main distinction criterion between CV-AVMs and other types of AVMs in DSA recordings.

Fig 7 summarizes all CA transport patterns in terms of the scaled arterio-venous delay t_d_ and the scaled absolute dispersivity of the intervening vasculature AD, which clearly shows an accumulation of t_d_ and AD at high values for CV-AVMs and at lower values for type IV lesions, independent of interpatient variability of other factors. If the malformations only contain small changes to the physiological reference configuration, AD and t_d_ are in a similar range as the baseline network models. As expected, *in-vivo* and computational type IIIb and II AVMs both show an even faster and less dispersive CA shunting than most type IV lesions. In all cases, computational results agree with their corresponding patient cases.

These new insights into microvascular AVM angioarchitectures may pave the way for more efficient diagnostic processes as well as safer and more specific interventional treatment options, reducing the patient’s exposure to contrast and radiation and most importantly, minimizing the incidence of unidentified and mistreated lesions. Further clinical integration of computational models will provide more powerful tools for precise disease identification and hemodynamic specification of vascular pathologies.
